# Nurse-facilitated preexposure prophylaxis delivery for adolescent girls and young women seeking contraception at retail pharmacies in Kisumu, Kenya

**DOI:** 10.1097/QAD.0000000000003447

**Published:** 2022-11-29

**Authors:** Jillian Pintye, Josephine Odoyo, Bernard Nyerere, Pauline Achieng, Evelyne Araka, Christine Omondi, Katrina F. Ortblad, Melissa L. Mugambi, Jared M. Baeten, Elizabeth A. Bukusi

**Affiliations:** a University of Washington, Seattle, Washington, USA; b Kenya Medical Research Institute, Kisumu, Kenya; c Fred Hutchinson Cancer Research Center, Seattle, Washington; d Gilead Sciences, Foster City, California, USA.

**Keywords:** adolescents, HIV prevention, preexposure prophylaxis, women

## Abstract

We evaluated preexposure prophylaxis (PrEP) uptake, initiation, and continuation within a nurse-facilitated pharmacy-based delivery model for Kenyan adolescent girls and young women (AGYW) seeking contraception at retail pharmacies.

**Methods:**

From October 2020 to March 2021, PrEP-trained nurses were stationed at three retail pharmacies in Kisumu, Kenya. AGYW (aged 15–24 years) purchasing contraception (emergency contraception, oral contraceptive pills, injectables, implants, condoms) were counseled on PrEP, completed HIV testing, and offered a free 1-month supply of PrEP pills per national guidelines by nurses under supervision of a remote physician. We evaluated uptake among all AGYW offered PrEP. At 30 days after uptake, we evaluated PrEP use initiation and plans for continuation.

**Results:**

We enrolled 235 AGYW clients who were HIV-negative and purchasing contraception at pharmacies. Emergency contraception was the most frequently purchased contraceptive (35%). Median age was 22 years (IQR 19–23), 44% were currently in school, and 33% currently had multiple sexual partners. One-fourth (24%) exchanged sex for money or favors and 14% had sex while intoxicated in the prior 6 months. Overall, PrEP uptake was 85%; at 1 month, 82% had initiated PrEP use and 68% planned to continue use. Among those initiating PrEP, 69% were willing to pay for PrEP at retail pharmacies (median KES 150, IQR 100–200) even if available for free at public sector facilities.

**Conclusion:**

In this evaluation of nurse-facilitated PrEP delivery at pharmacies in Kenya, a substantial proportion of AGYW who purchased contraception subsequently initiated PrEP, planned to continue use, and were willing to pay for PrEP.

## Introduction

HIV incidence remains unacceptably high for cisgender adolescent girls and young women (AGYW) in East and Southern Africa [1]. Preexposure prophylaxis (PrEP) HIV prevention tools are promising, with tenofovir (TFV)-based daily oral PrEP and the dapivirine vaginal ring recommended by the WHO for cisgender women at an increased risk for HIV acquisition. Kenya is a leader for PrEP delivery in Africa and efforts are ongoing to increase PrEP access with AGYW as a priority group [2–4]. Anchoring PrEP services to where AGYW access family planning offers an opportunity to efficiently reach AGYW with HIV acquisition risk. The PrEP Implementation for Young Women and Adolescents (PrIYA) Program demonstrated feasibility of integrated daily oral PrEP delivery for AGYW within family planning clinics in Kenya [2]. In the PrIYA program, a PrEP trained nurse was assigned to integrate PrEP into routine family planning services. PrEP uptake was 16% among AGYW when offered PrEP by nurses at family planning clinics [2]. Yet, up to 40% of Kenyan women access contraception outside of public health facilities [5], including at retail pharmacies, and would be missed by clinic-based PrEP platforms. Novel strategies to deliver PrEP are needed to reach AGYW who do not frequently attend clinics.

Retail pharmacies can increase options for reaching individuals with PrEP [6]. Individuals visit pharmacies for a variety of reasons, enabling those seeking PrEP to maintain privacy and reduce stigma associated with PrEP [7,8]. Retail pharmacies outnumber clinics and are typically located in high-traffic areas accessible to those who may be interested in PrEP [9–11]. This approach also expands the choice of PrEP access points. Several pilot and formative qualitative studies of pharmacy-based PrEP, mainly in Kenya and South Africa, consistently find that pharmacy-based PrEP is feasible, acceptable, and preferred among key populations [6,12–14]. To date, few studies of pharmacy-based PrEP delivery focus on AGYW, which may have unique considerations.

We adapted and evaluated a family planning clinic-based PrEP model and piloted nurse-facilitated PrEP, supported with HIV self-testing, for Kenyan AGYW seeking contraception at retail pharmacies.

## Materials and methods

### Study population

From October 2020 to March 2021, trained study nurses with experience providing contraception and PrEP to AGYW were stationed at three retail pharmacies in Kisumu, Kenya. All study nurses were cisgender women from the greater Kisumu region. Pharmacies less than 3 kilometers from a referral clinic were purposively selected based on having a large volume of AGYW clientele (e.g., near university dormitories), and having a private area for consultation. AGYW aged 15–24 years who purchased contraception [emergency contraception, oral contraceptive pills (OCPs), injectables, implants, condoms] at the pharmacy were eligible for enrollment. Study nurses enrolled eligible AGYW until 200 participants accepted PrEP pills.

### Study procedures

Per national guidelines for PrEP delivery in Kenya, study nurses delivered counseling, HIV testing, and PrEP pills to all eligible AGYW at the participating pharmacies under supervision of a remote physician. Nurses tested participants for HIV in the private consultation area using oral-fluid HIV self-tests. For participants who tested HIV-negative, nurses offered a 1-month free supply of daily oral PrEP. For participants who tested HIV-positive, PrEP was not dispensed and nurses referred them to the nearest clinic for confirmatory testing. Nurses provided counseling on topics raised by AGYW, including contraceptive types and issues related to relationships and navigating PrEP use. All participants (regardless of PrEP uptake) were offered two free oral HIV self-tests for at-home couples or partner testing.

### Data collection

Nurses administered a questionnaire to all participants during their pharmacy visit to collect information on participants’ demographics, contraceptive use, relationship characteristics, and sexual behaviors. We evaluated behaviors associated with HIV risk using a standardized tool used by the Kenya Ministry of Health to screen for PrEP eligibility that includes partners’ HIV status, condomless sex, engagement in transactional sex, experience of intimate partner violence (IPV), and being forced to have sex in the last 6 months [15].

All AGYW who were dispensed PrEP pills at enrollment were scheduled for a one-time follow-up visit after 30 days. Follow-up visits were conducted in-person at the pharmacy whenever possible or via telephone due to COVID-19 precautions. At 30 days after dispensation of PrEP pills, we evaluated self-reported PrEP use initiation (i.e., swallowing pills), plans for continuation of PrEP (among those who initiated), and use of dispensed HIV self-tests. Participants who planned to continue PrEP were referred to the nearest public facility providing PrEP.

### Statistical analysis

We used descriptive statistics to determine the frequency of demographic and relationship characteristics, pregnancy history and contraceptive use, behaviors associated with HIV risk, and PrEP and HIV self-test uptake and use. HIV risk behaviors were assessed using an empiric risk score validated to predict risk of HIV acquisition among young women in sub-Saharan African settings [16]. We utilized the modified version of this risk score (i.e., excluding having an STI since STI testing was unavailable) [16]. “High” HIV risk is defined by an HIV risk score of at least 5 (corresponding to 5–15% HIV incidence in cohorts of African women) [16]. Among AGYW who accepted PrEP at enrollment and completed a follow-up visit, we summarized PrEP use initiation and plans for continuation and HIV self-test use by male partners (as self-reported by AGYW). We compared PrEP and HIV self-test outcomes by behavioral HIV risk category using Chi-squared tests for categorical measures and Kruskal–Wallis tests for continuous measures. All analyses were conducted using Stata, version 15.0 (StataCorp, College Station, Texas, USA).

### Considerations for humans

The Kenya Medical Research Institute Scientific and Ethics Research Committee (SERU) and University of Washington Human Subjects Review Committee reviewed and approved the study.

## Results

In total, we enrolled 238 AGYW who purchased contraception at pharmacies (71% of eligible AGYW, Supplemental Material). Three AGYW tested HIV-positive after enrollment and were excluded from analyses; all completed referrals to the nearest health facility for confirmatory HIV testing and HIV treatment. Among AGYW that tested HIV negative (*n* = 235), the median age was 22 years (interquartile range [IQR] [19–23]), 18% were 18 years or less, 44% were in school, and few (5%) were formally employed (Table 1). Nearly half (48%) of AGYW reported having previously been pregnant. Emergency contraception was the most common contraceptive (35%) purchased by AGYW on the day of enrollment. One in three (33%) AGYW reported having previously used emergency contraception more than twice.

**Table 1:** Characteristics of HIV-negative adolescent girls and young women seeking contraception at retail pharmacies (*n* = 235).

	*N* (%) or median (IQR)
Demographic and clinical characteristics	
Age (years)	22.0 (19.0, 23.0)
Age category (years)	
15–18	42 (18%)
19–21	66 (28%)
22–24	127 (54%)
Relationship status	
Single, no partners at all	4 (2%)
Casual partner(s) only	8 (3%)
One primary partner only	146 (62%)
One primary partner and casual partners(s)	77 (33%)
Married	55 (23%)
Currently in school	104 (44%)
Type of school (*n* = 104)	
Primary school	2 (2%)
Secondary school	36 (35%)
Tertiary/College/University	66 (63%)
Completed years of education	12.0 (10.0, 13.0)
Formally employed	12 (5%)
Ever been pregnant	112 (48%)
Contraceptive purchased today	
Injectable	46 (20%)
OCP	30 (13%)
EC	82 (35%)
Condoms	64 (27%)
Implant	12 (8%)
Previously used emergency contraception more than twice	77 (33%)
Ever tested for HIV before today	226 (96%)

Relationship characteristics	
Currently has any sexual partner(s)	227 (97%)
Partner providers financial support	199 (88%)
Partner has other sexual partners	
No	46 (20%)
Yes	47 (21%)
Unsure	134 (59%)
Lives with partner	48 (21%)
Ever HIV tested with primary sexual partner	93 (41%)
Primary partners HIV status	
Negative	99 (44%)
Positive	1 (0.4%)
Unknown	127 (56%)
Partner age (years)	27.0 (23.0, 30.0)

Behavioral HIV risk factors (last 6 mos. unless indicated)	
Had sex without condom	230 (98%)
Had sex while being intoxicated	32 (14%)
Exchanged sex for money/favors	57 (24%)
Diagnosed or treated for an STI	14 (6%)
Forced to have sex against will	28 (12%)
Experienced intimate partner violence	32 (14%)
Drank any alcohol in the last 30 days	65 (28%)
HIV risk score ≥ 5^a^	155 (66%)

EC, emergency contraception; OCP, oral contraceptive pills; STI, sexually transmitted infection.

a We evaluated behavioral HIV risk using the Balkus *et al.*^19^ HIV risk scoring: age <25 years=2 points; unmarried or not living with partner=2 points; partner does not provide financial or material support=1 point; primary partner has other partners (yes or do not know)=2 points; alcohol use in the past 3 months=1 point. Scores of ≥5 correspond to 5–15 incident HIV cases per 100 person-years in cohorts of African women; risk scores of ≤4 correspond to <5 incident HIV cases per 100 person-years.

Almost all (99%) participants reported at least one behavior on the Ministry of Health's PrEP eligibility criterion (66% when excluding condomless sex as a criterion). In addition, 66% had an HIV risk score of at least 5 [16]. Almost half (47%) of participants had at least one PrEP eligibility criterion (excluding condomless sex) and also had an HIV risk score of at least 5. One-third (33%) of AGYW reported multiple sexual partners and over half (59%) were unsure if their male partners had other partners. Over half (56%) of participants also reported having a partner of unknown HIV status. One in four participants (24%) reported exchanging sex for money or favors and 14% had sex while intoxicated in the prior 6 months; 12% reported being forced to have sex against their will and 14% experienced IPV (Table 1).

### Preexposure prophylaxis and HIV self-test acceptance

All AGYW that tested HIV-negative were offered PrEP and 85% (*n* = 200) accepted PrEP and were dispensed a 30-day supply of PrEP pills. PrEP uptake was slightly higher among AGYW with high behavioral HIV risk compared with those with low risk (90% vs. 80%, *P* = 0.40, Table 2). The most frequent reasons for PrEP acceptance were feeling at risk for HIV (92%) and thinking a partner has other partners (84%). Overall, 71% accepted HIV self-test kits for at-home couples or partner testing and the most common reason for declining was fear of partner reactions (53%).

**Table 2:** Preexposure prophylaxis and HIV self-test kit acceptance and use among HIV-negative adolescent girls and young women seeking contraception at retail pharmacies (*n* = 235), by behavioral HIV risk^a^

	*N* (%) of Median (IQR)			
PrEP and HIV self-test acceptance outcomes *(n* *=* 235)	Overall (*n* = 235)	Low HIV risk^a^ (*n* = 80)	High HIV risk^a^ (*n* = 155)	*P* ^b^
Accepted PrEP pills	200 (85%)	64 (80%)	139 (90%)	0.040^∗^
Reason(s) PrEP not accepted (not mutually exclusive, *n* = 35)				
Low HIV risk perception	24 (75%)	13 (81%)	11 (69%)	0.414
Partner known HIV-negative	17 (53%)	7 (44%)	10 (63%)	0.288
Pill burden	17 (53%)	5 (31%)	12 (75%)	0.013^∗^
Fear of partners’ negative reactions	11 (34%)	6 (38%)	5 (31%)	0.710
Fear of side effects	4 (13%)	1 (6%)	3 (19%)	0.285
Fear of IPV	2 (6%)	2 (13%)	–	–
Reason(s) PrEP accepted (not mutually exclusive, *n* = 200)				
Feels at risk for acquiring HIV	186 (92%)	58 (91%)	128 (93%)	0.602
Thinks partner may have other partners	170 (84%)	46 (72%)	124 (89%)	0.002^∗^
Partner HIV status unknown	137 (68%)	42 (66%)	95 (68%)	0.701
Partner known HIV-positive	1 (1%)	–	1 (1%)	–
Other	11 (6%)	–	11 (8%)	–
Accepted HIV self-test kits	167 (71%)	59 (74%)	108 (70%)	0.514
Reason(s) HIV self-test kit declined (*n* = 68)				
Fears partner reaction	36 (53%)	9 (43%)	27 (57%)	0.100
Knows partner(s) is HIV-negative	28 (41%)	10 (48%)	18 (38%)	
Knows partner(s) is HIV-positive	2 (3%)	–	2 (4%)	–
Other	2 (3%)	2 (10%)	–	–

PrEP use outcomes (*n* = 200)				
Completed follow-up visit at 30 days postacceptance of PrEP pills	188 (94%)	60 (94%)	128 (92%)	0.169
Swallowed PrEP pills since PrEP acceptance (*n* = 188)	155 (83%)	46 (77%)	109 (85%)	0.154
When was PrEP first swallowed after accepting pills (*n* = 155)				
Same day	100 (65%)	28 (61%)	72 (66%)	0.622
>1 day to 1 week	46 (30%)	16 (35%)	30 (28%)	
>1 week to 2 weeks	9 (6%)	2 (4%)	7 (6%)	
Reason(s) for not swallowing PrEP pills (not mutually exclusive, *n* = 33)				
Have not had sex in the last month	12 (36%)	4 (29%)	8 (42%)	0.424
Relationship ended	6 (18%)	4 (29%)	2 (11%)	0.184
Did not feel at risk of HIV	15 (45%)	5 (36%)	10 (53%)	0.335
Partner used self-test and was HIV-negative	12 (36%)	5 (36%)	7 (37%)	0.947
Planning to start PrEP later	1 (3%)	–	1 (5%)	–
Other	5 (15%)	–	5 (26%)	–
Plans to continue PrEP use (*n* = 155)				
No	41 (27%)	10 (22%)	31 (28%)	0.561
Yes	105 (68%)	34 (74%)	71 (65%)	
Unsure	9 (6%)	2 (4%)	7 (6%)	
Reason(s) for discontinuing PrEP use (not mutually exclusive, *n* = 41)				
Experienced side effects	21 (51%)	5 (50%)	16 (52%)	0.545
Partner used self-test and was HIV-negative	12 (37%)	3 (30%)	9 (29%)	0.755
Relationship ended/have not seen partner	9 (22%)	1 (10%)	8 (26%)	0.263
Other	2 (5%)	–	2 (6%)	–

Willingness to pay for PrEP at pharmacy				
Willing to pay for PrEP, even if available for free at public clinics (*n* = 155)	107 (69%)	28 (61%)	79 (73%)	0.153
Maximum amount willing to pay for a one-month supply of PrEP (KES, *n* = 107)^c^	150 (100–200)	150 (100–200)	150 (100–300)	0.346
Willing to pay the following amount for PrEP (not mutually exclusive, *n* = 107)				
KES <100	94 (88%)	26 (93%)	68 (86%)	0.172
KES 100–500	74 (69%)	21 (75%)	53 (67%)	0.786
KES 500–1000	3 (3%)	1 (4%)	2 (3%)	0.338
KES >1000	0 (0%)	–	–	–
Reason(s) why willing to pay for PrEP at pharmacy (not mutually exclusive, *n* = 107)				
Convenience	106 (99%)	27 (96%)	79 (100%)	0.091
Anonymity	64 (60%)	15 (54%)	49 (62%)	0.433
Less stigma	91 (85%)	22 (79%)	91 (85%)	0.263
More comfortable	104 (97%)	27 (96%)	77 (97%)	0.775
No wait times	100 (94%)	25 (89%)	75 (95%)	0.299
Closer to my home	97 (91%)	26 (93%)	71 (90%)	0.641
Other	5 (5%)	1 (4%)	4 (5%)	0.702

HIV self-test outcomes				
Accepted HIV self-test kits at enrollment visit (*n* = 188)	133 (71%)	47 (78%)	86 (67%)	0.117
Partner used HIV self-test (*n* = 133)	101 (76%)	35 (74%)	66 (77%)	0.769
Used HIV self-test together with partner (*n* = 101)	97 (96%)	33 (94%)	64 (97%)	0.510
Partner HIV self-test results (*n* = 101)				
Negative	99 (98%)	34 (97%)	65 (98%)	0.298
Positive	1 (1%)	–	1 (2%)	–
Do not know	1 (1%)	1 (3%)	–	–
Experienced harm or negative reactions from partners regarding the self-test (*n* = 133)	10 (8%)	2 (4%)	8 (9%)	0.291

∗ *P* < 0.05.

a We evaluated behavioral HIV risk using the Balkus *et al.*^ 19^ HIV risk scoring: age <25 years = 2 points; unmarried or not living with partner = 2 points; partner does not provide financial or material support = 1 point; primary partner has other partners (yes or do not know) = 2 points; alcohol use in the past 3 months = 1 point. Scores of ≥5 correspond to 5–15 incident HIV cases per 100 person-years in cohorts of African women; risk scores of ≤4 correspond to <5 incident HIV cases per 100 person-years.

b Chi-squared tests for categorical measures and Kruskal-Wallis tests for continuous measures.

c In response to ‘How much would you be willing to pay for a one-month supply of PrEP if purchased from a retail pharmacy (this includes HIV testing and counseling services)?’.

### Preexposure prophylaxis initiation and HIV self-test use

Among 200 AGYW who accepted PrEP pills, 188 (94%) completed a follow-up visit at 1 month; nurses were unable to contact the remaining 6% (*n* = 12). At 1 month, 82% (155/188) who accepted PrEP at enrollment reported initiating PrEP use, of which 68% (105/155) planned to continue PrEP and were referred to the nearest PrEP-dispensing health facility. Among those who accepted PrEP at enrollment but did not initiate use by 1 month (*n* = 33), reasons for not initiating included not feeling at risk of HIV (45%), not having sex in the last month (36%), and male partners testing HIV-negative with the self-test kits dispensed at enrollment (36%). Among those who initiated PrEP use (*n* = 155), 65% swallowed PrEP pills the same day they were dispensed. Over two-thirds (69%) of those who initiated PrEP use were willing to pay for PrEP pills at retail pharmacies even if PrEP pills are available for free at public sector facilities. Frequent reasons for willingness to pay for PrEP included that PrEP delivery at retail pharmacies was convenient, comfortable, and anonymous, had no wait times and less stigma, and was closer to home as compared to public sector health facilities (Table 2). The maximum amount AGYW were willing to pay for a 1-month supply of PrEP was USD 1.50 (IQR 1.00–2.00).

Among AGYW who completed follow-up visits and previously accepted HIV self-test kits (*n* = 133), 76% (101/133) had partners who used an HIV self-test and 96% used an HIV self-test together with their partner. One AGYW reported that their partner tested HIV-positive and was on HIV treatment. Overall, 10 (7.5%) AGYW who previously accepted HIV self-test kits at enrollment reported experiencing harm or negative reactions from partners regarding the self-tests. Harms experienced included partners being upset (i.e., no violence, *n* = 8), verbal abuse (*n* = 2), emotional abuse (e.g., isolation, *n* = 1), and physical violence (*n* = 1).

## Discussion

In this evaluation of nurse-facilitated PrEP provision at retail pharmacies in Kenya, over half of AGYW who were offered PrEP subsequently initiated PrEP use and planned to continue use, and a substantial proportion were willing to pay for PrEP at pharmacies. Our results also suggest that AGYW seeking contraception at pharmacies may more frequently have behaviors associated with HIV risk and also self-select PrEP when offered compared with AGYW who seek contraception at family planning clinics. To our knowledge, this pilot study was the first evaluation of pharmacy PrEP delivery tailored to AGYW.

Recent studies consistently find that pharmacy-based PrEP is feasible, acceptable, and preferred among various populations [6,12–14]. In our study among AGYW seeking contraception, we found much higher PrEP uptake (85%) at retail pharmacies than other PrEP demonstration projects among Kenyan AGYW in family planning clinics (range 4–22%) [2,17–19]. Our findings suggest that pharmacies are a potentially high-demand PrEP access point for AGYW. Low HIV risk perception was the most frequent reason for declining PrEP (75%), which could be addressed through enhancing counseling about HIV risk [20–22] or incorporating STI testing into PrEP counseling as an empiric marker for HIV risk [23]. In our study, 68% of AGYW who initiated PrEP planned to continue use at 1 month, which is higher than AGYW who initiated PrEP within family planning clinics in Kisumu (41%) [2]. Future studies of pharmacy-based PrEP delivery for AGYW that incorporate longitudinal follow-up and objective adherence measurement are required to understand longer-term PrEP outcomes in this setting.

We found higher frequency of having multiple sexual partners, forced sex, IPV, alcohol use, and exchanging sex for money/gifts among AGYW seeking contraception at retail pharmacies compared with our prior work among AGYW in family planning clinics [2,17]. Overall, 66% of AGYWs seeking contraception at pharmacies were at a high risk of acquiring HIV based on having an HIV risk score of at least 5 [16], compared with 24% in a prior study of those seeking contraception at family planning clinics [17]. Similar to prior studies, our results suggest that AGYW concerned about avoiding or postponing pregnancy are also at risk for HIV [24]. Our data support the urgency to strengthen integration of family planning services with combination HIV prevention, including PrEP provision.

Few studies have evaluated HIV self-test distribution with PrEP among nonpregnant women in family planning settings, though existing data suggest acceptance of self-tests is not associated with PrEP uptake within family planning clinics [25]. We found that some AGYW who accepted PrEP pills and HIV self-tests decided not to initiate PrEP use or discontinued use after partners tested HIV-negative using self-tests. These findings suggest that HIV self-tests could be a tool for guiding appropriate PrEP decisions among AGYW and increasing awareness of male partner HIV status, though our sample size was limited. In our prior study of HIV self-test distribution in family planning clinics in Kisumu, experience of harm because of the self-tests was reported by 7% of women [25], which is very similar to the frequency of harm reported in our pilot. More research is needed to evaluate the cost and utility of co-dispensing HIV self-tests with PrEP for AGYW within pharmacy settings.

Our study has limitations. We are unable to assess the contribution of nurse-navigators to PrEP and HIV self-test decision-marking among AGYW in this study. We can only summarize planned PrEP continuation self-reported by AGYW. We also relied on self-report for HIV self-testing outcomes among male partners, similar to other studies of HIV self-test secondary distribution in Kenya [25,26]. We did not ascertain PrEP adherence data as our pilot was primarily focused on PrEP acceptance and initiation. Future evaluation of longitudinal PrEP use and adherence are needed to fully elucidate PrEP outcomes among AGYW in this setting.

In conclusion, nurse-facilitated PrEP provision at pharmacies was feasible, reached AGYW with behaviors associated with HIV risk, and resulted in high PrEP initiation rates among AGYW seeking contraception. A substantial proportion of AGYW who purchased contraception subsequently initiated PrEP, planned to continue PrEP use, and were willing to pay for PrEP at pharmacies. In addition, co-dispensing HIV self-tests with PrEP guided PrEP decision-making. Pharmacy-based PrEP delivery platforms may be an important PrEP access point for AGYW seeking to prevent pregnancy and HIV acquisition.

## Acknowledgements

J.P., J.M.B., and E.B. conceived and designed the study. J.O., B.N., P.A., E.A., and C.O. conducted data collection. J.P. and B.N. analyzed the data. J.P. and E.B. drafted the article. All authors critically reviewed the manuscript. The authors thank all the study participants for their contributions and the staff at all participating institutions for their support. The study team acknowledges the Director of KEMRI for support.

This study was funded by the Bill & Melinda Gates Foundation (INV-008628) and the *Eunice Kennedy Shriver* National Institute of Child Health & Human Development (NICHD, R01HD108041) with support from the University of Washington Center for AIDS Research (P30 AI027757). J.P. was additionally supported by NICHD (R01HD100201) and the National Institute of Nursing Research (R01NR019220). K.F.O. was additionally supported by the National Institute of Mental Health (R00MH121166, R34MH120106). M.L.M. was additionally supported by NIMH (K01MH122326).

### Conflicts of interest

J.M.B. is an employee of Gilead Sciences, outside of the present work. The remaining authors have no financial conflicts of interest to declare.
